# Recent advances in the clinical management of autosomal dominant polycystic kidney disease

**DOI:** 10.12688/f1000research.17109.1

**Published:** 2019-01-29

**Authors:** Roser Torra

**Affiliations:** 1 Inherited Renal Disorders, Nephrology Department, Fundació Puigvert, REDINREN, IIB Sant Pau, Universitat Autònoma de Barcelona, Barcelona, 08025, Spain

**Keywords:** ADPKD, diagnosis, management, treatment, prediction

## Abstract

Autosomal dominant polycystic kidney disease (ADPKD) is a genetic systemic disorder causing the development of renal and hepatic cysts and decline in renal function. It affects around 1 in 1,000 live births. Early hypertension and progressive renal failure due to massive enlargement of cysts and fibrosis are hallmarks of the disease. This article reviews recent advances in ADPKD and focuses mainly on diagnosis, management, and prediction of the course of the disease.

## Introduction

Autosomal dominant polycystic kidney disease (ADPKD) is the most common monogenic cause of kidney failure, occurring in more than 1 in 400 to 1,000 live births and accordingly representing a major socioeconomic medical problem globally ^1^. ADPKD arises owing to mutations in the *PKD1* gene (about 85% of cases) or the *PKD2* gene (about 15% of cases), which encode the proteins polycystin-1 (PC1) and -2 (PC2), respectively ^2, 3^. Compared with patients who have *PKD1* mutations, those with *PKD2* mutations have a milder phenotype and reach end-stage renal disease (ESRD) about 20 years later ^4– 6^. ADPKD is characterized by progressive development and enlargement of cysts in all nephron segments but preferentially in the collecting duct, and ESRD occurs in 50% of patients by the age of 60 years ^1^. It is associated with cyst formation in other organs, primarily the liver and pancreas, and with cardiovascular anomalies such as intracranial arterial aneurysms and vascular dissections. Huge variation is observed in disease phenotype and progression, even within families; the reason for this is poorly understood, but genetic modifiers, including hypomorphic alleles, probably play a significant role.

Although a definitive curative treatment is not currently available, there is a disease-modifying therapy using a V2 receptor antagonist (tolvaptan) ^7, 8^. This article guides the reader through recent achievements, and the clinical consequences, within the field of ADPKD.

## Genetics and genetic testing

Mutations in other genes, such as *GANAB* and *DNAJB11*, have recently been identified in a small subgroup of patients ^9, 10^, but the phenotypes related to these mutations are not exactly the same as those caused by mutations in *PKD1* or *PKD2*. This may suggest that, instead of using the name “ADPKD”, it would be appropriate to name these diseases after the mutated gene, as has been done, for example, for ADTKD ^11^. Whereas mutations in *GANAB* give rise to a mild form of cystic disease that does not progress to ESRD ^9^, mutations in *DNAJB11* cause cystic disease in the context of which ESRD may develop in the absence of marked kidney enlargement ^10^.

Genetic testing has undergone remarkable progress, as is very evident if one considers the case of ADPKD. When the *PKD1* and *PKD2* genes were first identified, genetic testing was performed by means of linkage analysis ^5^. Not only was this time consuming but also, more importantly, several affected and non-affected family members were needed in order to identify the risk haplotype. As soon as the *PKD1* gene was identified, it became evident that genetic testing for the gene would be burdensome owing to the large transcript size (>14 kb) and reiteration of the genomic area encoding about 75% of the protein on the same chromosome ^12^. The group of Harris then developed a technique that used long-range polymerase chain reaction and benefited from the few non-repeated nucleotides ^13^. Although this allowed diagnosis of a single individual, it remained time consuming and was also expensive. Over the past 3 years, next-generation sequencing (NGS) has changed the scenario ^14– 16^. The use of NGS for genetic diseases such as ADPKD has reduced costs, improved sensitivity, and reduced turnaround time ^17, 18^. A further advantage of this relatively new technique is that gene panels or whole exome sequencing can be used, allowing the detection of mutations in ultrarare genes that account for a very small number of cases of renal cystic disease ^17^. The drawback of testing *PKD1* is the huge number of variants of unknown significance (VUSs) that can be identified, around 10 per patient ^18^. Some of these VUSs have previously been published as mutations, but for many pathogenicity is still to be elucidated ^19^. This is of the utmost relevance when a patient seeks preimplantation genetic diagnosis (PGD) or when a disease-modifying treatment for ADPKD can be offered but there is uncertainty over the diagnosis. PGD has evolved over recent years to become a valuable technique to offer patients with severe genetic diseases, although there are ethical issues regarding its use for non-severe genetic diseases. ADPKD is halfway between these categories, and genetic counseling is highly recommended to ensure sound understanding of the pros and cons of such a procedure ^20^. Probably, each case or family should be counseled differently according to the severity of the disease, but patient attitudes to PGD vary considerably depending on how well the family is dealing with the disease, religious aspects, age, disease severity, and so on.

## Progression of autosomal dominant polycystic kidney disease

A large number of factors have been identified as influencing disease progression ^21^, and whereas some are modifiable, many are not ^22^. Among the latter are male sex, *PKD1* mutations (worse if protein-truncating (PT)), early development of renal symptoms, early detection of high blood pressure ^23^, and large kidney volumes in relation to age and height ^24, 25^. Modifiable factors that should be highlighted in patients with ADPKD include smoking; blood pressure; lipid levels; water, protein, and calorie intake; and body mass index (BMI) ^26– 31^. Also, some biomarkers, such as MCP-1, FGF23, and copeptin, have been found to be predictive factors of ADPKD progression ^32– 34^.

The prediction of which patients will have a rapid progression is crucial not only for the decision of whether to use a disease-modifying drug but also for the recruitment of patients to clinical trials ^35^. Although in the past few years many attempts have been made to provide tools predictive of rapid disease progression, to date there is no gold standard. Some of these predictive tools employ imaging; an example is the Mayo Clinic ADPKD calculator, which uses the height-adapted total kidney volume (htTKV) to classify patients into one of five classes (A–E) on the basis of growth rates: less than 1.5%, 1.5–3%, 3–4.5%, 4.5–6%, or more than 6% per year ^36^. Measuring TKV for use as a prognostic biomarker does not require high precision. Measurement by the ellipsoid equation as well as by means of various imaging modalities is possible ^27, 37^. Another imaging approach that can be used to predict PKD progression involves the measurement of renal length. Bhutani *et al*. demonstrated that a renal diameter of 16.8 cm is predictive of chronic kidney disease (CKD) stage 3 within 8 years ^38^. An alternative to imaging-based prognostic strategies is the Predicting Renal Outcome in Polycystic Kidney Disease (PROPKD) scoring system, as shown in a large cohort from Brittany, France ^23^. This scoring system uses both clinical and genetic data to stratify risk of disease progression. Patients may reach a score suggestive of rapid progression if they have hypertension or early urinary symptoms before the age of 35 years plus a PT mutation. Male sex is also included in the scoring system. Obvious drawbacks of this predictive tool are the need to perform genetic testing, which may not be available in many centers, and the impossibility of employing it in patients younger than 35 who are asymptomatic. In addition, it is subject to the need to establish a relationship between early symptoms and ADPKD and the limitation that many rapid progressors with PT mutations do not develop early symptoms.

The European Renal Association-European Dialysis and Transplant Association (ERA-EDTA) Working Groups on Inherited Kidney Disorders and European Renal Best Practice (WGIKD/ERBP) developed a hierarchical decision-making algorithm that may offer guidance on the identification and prediction of rapid disease progression in patients with ADPKD and subsequently identify candidates for tolvaptan treatment ^39^. When this algorithm was tested in a cohort of 305 patients, it was found that 15.7% fulfilled the ERA-EDTA criteria and that the overall proportion of patients with rapid progression rose to 27% upon incorporation of expanded criteria based on data from the REPRISE (Replicating Evidence of Preserved Renal Function: an Investigation of Tolvaptan Safety and Efficacy in ADPKD) trial ^40^ (that is, age of less than 56 years and estimated glomerular filtration rate [eGFR] of more than 30 mL/min/m ^2^) ^41^. This study, together with a recent assessment of an unpublished large series of patients, calls into question the suitability of the algorithm, especially in its first steps when limiting for age/eGFR and using retrospective eGFR. Each country, even region, in Europe has either approved the use of the drug on these terms or proposed some changes. For example, the National Institute for Health and Care Excellence (NICE) decision in the UK was to treat on the basis of these criteria but only patients with CKD stage 2 to 3 without limiting age, whereas in Scotland they approved its use in stage 1. In the US, the preferred approach for definition of a rapid progressor is based on htTKV, age, and eGFR, basically using the Mayo ADPKD calculator ^42^. Risk assessment in ADPKD is an evolving process that will undergo further refinement as new clinical data are obtained and prediction tools are developed. The performance of risk assessment guidelines needs to be evaluated and validated by real-life clinical data, but it is probable that the use of a composite tool including age, renal function, imaging, family history and possibly genetic testing will be able to define rapid progression very accurately.

## Basic renal protective measures

Based on the HALT-PKD (Halt Progression of Polycystic Kidney Disease) trial, the recommendations regarding blood pressure control for ADPKD were recently lowered ^26^. The target should now be 95–110/60–75 mmHg in 15- to 49-year-old patients with an eGFR of more than 60 mL/min/1.73 m ^2^. First-line management of hypertension should include a blocker of the renin–angiotensin–aldosterone system (angiotensin-converting enzyme inhibitors and angiotensin-II receptor blockers) ^27^. Second- and third-line treatments should be diuretics and beta-blockers. Calcium channel blockers (particularly the dihydropyridine class) should be considered if blood pressure is not controlled by the other agents ^27^. The CRISP (Consortium for Radiologic Imaging Studies of Polycystic Kidney Disease) and HALT studies suggest that moderate dietary sodium restriction (2.3–3 g) be recommended to patients with ADPKD ^22^.

In patients with cyst infection, a positron emission tomography/computed tomography scan is recommended to enable determination of the location of cysts for the purpose of diagnosis or drainage ^43^. Fluoroquinolones remain the first-line antibiotic treatment, but their side effects and the increasing prevalence of fluoroquinolone-resistant Gram-negative bacilli mean that cyst infections frequently represent a very large burden.

Until more information becomes available ^44, 45^, moderate enhancement of hydration, spread out over the course of 24 hours, is recommended with the goal of maintaining an average urine osmolality of not more than 280 mOsm/L ^27, 46^.

Based on experimental studies and lessons learned from CKD in general, a daily protein intake of 0.8–1.0 g/kg ideal body weight seems appropriate in ADPKD ^27^. Although protein-restricted diets are usually already low in phosphorus, patient education on the need to avoid processed foods that contain readily absorbed inorganic phosphates is appropriate. Sodium bicarbonate supplementation is recommended in order to treat metabolic acidosis, and the aim is to maintain the plasma bicarbonate level at a minimum of 22 mmol/L ^27^.

Mild to moderate food restriction has been found to markedly reduce cystogenesis in animal models of PKD ^47, 48^. This fact, together with evidence that excess food intake has a detrimental effect both in PKD trials and in CKD in general, means that moderation of caloric intake and avoidance of an above-average BMI should be recommended to patients with ADPKD ^29^.

A small trial with statins in children has yielded positive results ^49^, but a post-hoc analysis of the HALT-PKD trials failed to demonstrate a benefit of statin therapy on renal outcomes ^50^. However, evidence in ADPKD ^22, 31^, and in CKD in general, indicates that it is appropriate to keep the low-density lipoprotein (LDL) cholesterol level at not more than 100 mg/dL.

## Disease-modifying treatments

In the TEMPO (Tolvaptan Efficacy and Safety in Management of Autosomal Dominant Polycystic Kidney Disease and Its Outcomes) 3:4 trial, the vasopressin receptor antagonist tolvaptan was shown to slow the growth of cystic kidneys and the deterioration of renal function ^7^. Based on these results, the European Medicines Agency in 2015 approved tolvaptan to slow the progression of cyst development and renal insufficiency in ADPKD in adults with CKD stages 1–3 at initiation of treatment and evidence of rapidly progressing disease ^51^. TEMPO 4:4 showed that benefit is sustained over time ^52^.

In 2018, tolvaptan was approved by the US Food and Drug Administration for the purpose of slowing decline in kidney function in adults at risk of rapidly progressing ADPKD. This decision was based on additional information provided by the REPRISE trial, which demonstrated slowing in the deterioration of renal function, even in the later stages of renal failure ^8^.

Currently, more than 6,000 patients with ADPKD are being treated with tolvaptan around the world. However, tolvaptan does have side effects. It markedly impairs urinary concentrating ability and therefore patients experience polyuria, nocturia, and polydipsia ^7, 8^. In addition, a small proportion of patients develop liver function abnormalities; it appears that whereas some of these abnormalities can be significant, all resolve upon drug discontinuation ^53^. This rare drug-induced liver injury led to the institution of a risk evaluation and mitigation strategy with monthly liver enzyme tests for the first 18 months and then at 3-month intervals.

**Figure 1.**
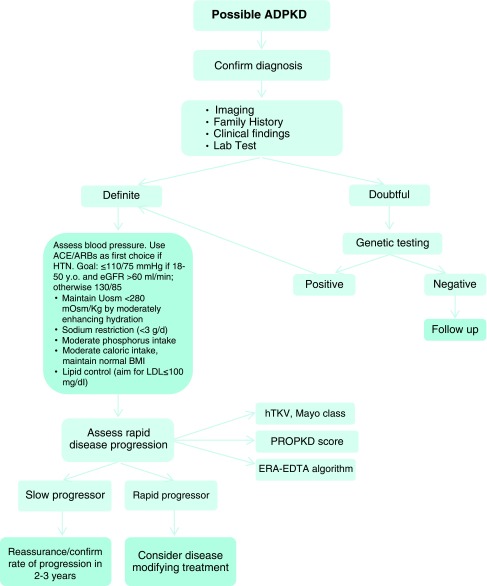
Diagnosis and management of autosomal dominant polycystic kidney disease. ACE/ARB, angiotensin-converting enzyme/angiotensin receptor blocker; ADPKD, autosomal dominant polycystic kidney disease; BMI, body mass index; eGFR, estimated glomerular filtration rate; ERA-EDTA, European Renal Association-European Dialysis and Transplant Association; hTKV, height-adapted total kidney volume; HTN, hypertension; LDL, low-density lipoprotein; PROPKD, Predicting Renal Outcome in Polycystic Kidney Disease; Uosm, urine osmolality. Modified from Chebib *et al*. ^27^ and Chebib *et al*. ^42^.

A few other drugs, such as mammalian target of rapamycin (mTOR) inhibitors, somatostatin analogues, and bosutinib, also underwent clinical trials but failed to show any positive effect on disease progression ^54– 57^. Recommendations for the management of ADPKD are shown in Figure 1.

Many new drugs are being tested, and it is probable that more than one drug will be needed to target the different abnormal pathways found in PKD cells.
